# A benchmark for machine-learning based non-invasive blood pressure estimation using photoplethysmogram

**DOI:** 10.1038/s41597-023-02020-6

**Published:** 2023-03-21

**Authors:** Sergio González, Wan-Ting Hsieh, Trista Pei-Chun Chen

**Affiliations:** AI Center, Inventec Corporation, Taipei, 111 Taiwan

**Keywords:** Medical research, Biomarkers

## Abstract

Blood Pressure (BP) is an important cardiovascular health indicator. BP is usually monitored non-invasively with a cuff-based device, which can be bulky and inconvenient. Thus, continuous and portable BP monitoring devices, such as those based on a photoplethysmography (PPG) waveform, are desirable. In particular, Machine Learning (ML) based BP estimation approaches have gained considerable attention as they have the potential to estimate intermittent or continuous BP with only a single PPG measurement. Over the last few years, many ML-based BP estimation approaches have been proposed with no agreement on their modeling methodology. To ease the model comparison, we designed a benchmark with four open datasets with shared preprocessing, the right validation strategy avoiding information shift and leak, and standard evaluation metrics. We also adapted Mean Absolute Scaled Error (MASE) to improve the interpretability of model evaluation, especially across different BP datasets. The proposed benchmark comes with open datasets and codes. We showcase its effectiveness by comparing 11 ML-based approaches of three different categories.

## Introduction

Hypertension increases the risk of stroke, renal dysfunction, and other diseases, making it a primary cause of millions of deaths in the United States^1^. The frequent absence of symptoms leads to underdiagnosis while the condition can be controlled and treated once high blood pressure is identified^2^. Blood Pressure (BP) monitoring devices are categorized into two types depending on the usage scenarios: invasive and non-invasive devices. The invasive BP monitoring approach—even though a gold standard—requires arterial cannulation and can lead to serious complications. Whereas, non-invasive BP monitoring devices such as sphygmomanometers cannot monitor BP continuously since it is unrealistic to constantly perform cuff inflations and deflations. Alternatively, photoplethysmography (PPG), a small and portable optical device that continuously measures volumetric variations of blood circulation, provides a potential alternative to not only monitor the BP non-invasively but also to monitor it continuously.

PPG devices have long been used to measure heart rate and blood oxygen saturation levels^3,4^ due to their affordable price and portability. Many studies have shown interest in extending the use of PPG to BP monitoring^5–7^. Among them, Pulse Transit Time (PTT) based methods^5^, which require two PPG sensors, are considered classic with their simple algebraic inverse relation between PTT and BP. However, these methods require subject-specific calibration of the two waveforms from the two sensors. Single PPG approaches, on the other hand, are desirable as they would not require calibration. In recent years, Machine Learning (ML) and Deep Learning (DL) based BP estimation using the PPG has been growing in popularity^7–9^. Moreover, as PPG and Arterial Blood Pressure (ABP) are both continuous waveforms, it is possible to leverage the morphological similarity between them to estimate continuous BP^10^.

**Fig. 1:**
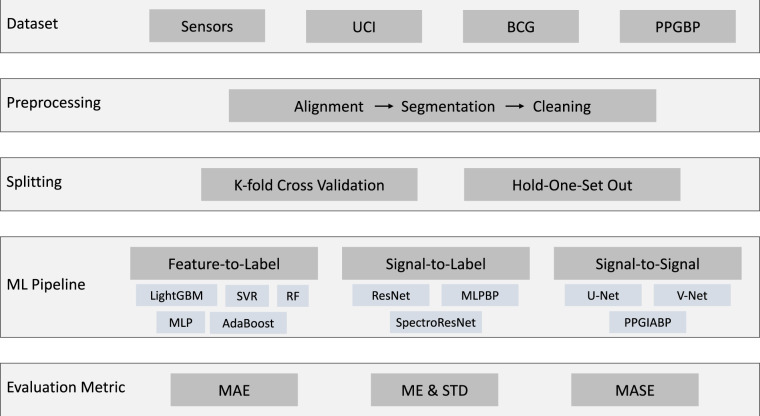
The flowchart of our proposed benchmark.

To assess the rise of many ML based BP estimation approaches using PPG, a benchmark is needed. Common caveats when comparing different approaches include using a dataset with specific characteristics or data distribution; differing pre-processing steps; invalid training and validation set splits^11,12^; and incomparable results due to different evaluation metrics^7,13^. In this paper, we propose a benchmark to properly compare different data-driven ML based BP estimation approaches as illustrated in Fig. 1. Such a benchmark is proposed with representative categories of ML & DL models in mind and works with four standard datasets^14^: Sensors^12^, UCI^15^, BCG^16^, and PPGBP^17^. First, we collect four publicly available datasets. They contain a large variety of data per subject, BP distributions, and data continuity characteristics i.e. recorded continuously or at different periods. Next, we streamline the preprocessing steps. Then we propose a validation strategy that not only preserves the data distribution among training, validation, and test sets but avoids subject information leaks among them. Furthermore, the ML pipeline in this benchmark is general enough to evaluate different categories of ML models to estimate intermittent or continuous forms of BP. We include three categories of algorithms according to the types of input and output: Feature-to-Label (Feat2Lab) includes models that take PPG features as input to generate discrete BP values, or labels, as output; Signal-to-Label (Sig2Lab) includes models that take continuous PPG waveforms as input to generate discrete BP values as output; and Signal-to-Signal (Sig2Sig) includes models that generate continuous ABP signal from continuous PPG signal. Lastly, to quantify the performance of different ML models, proper evaluation metrics are needed. Besides the BP standard metrics, we propose using Mean Absolute Scaled Error (MASE) as the BP evaluation measure. MASE was originally designed to assess the accuracy of forecasts with desirable properties, such as scale invariance and interpretability^18^. In BP estimation, MASE eases model comparison across different datasets, regardless of the BP range.

Our main contribution in this work is a benchmark for researchers to fairly compare different ML based BP estimation approaches. In our experiments, we show an extensive comparison of 11 state-of-the-art models of the aforementioned categories on four different datasets. For the family Feat2Lab, we consider the most popular PPG features^6,7,19^ and five successful ML algorithms. As for Sig2Lab, we include three DL automatic feature extractors of the PPG state-of-the-art^11,20,21^. While for Sig2Sig, we examine another three recently published approaches for PPG-to-ABP translation^12,22,23^. We first compare the models within each category and among the best of each category. Then, we analyze the most useful PPG’s features selected by Feat2Lab algorithms. Besides, we propose a proper validation strategy considering skewed BP distribution and subject information leaking, and we empirically show the impact of overlooking these considerations. Furthermore, we adapt MASE to best evaluate BP models among different datasets. We also share the processed datasets and their partitions^14^ and codes of data preparation and of the different algorithms with this paper. Given the benchmark, we share our insights on ML based BP estimation approaches, and hope to help propel the development of non-invasive BP estimation approaches forward.

## Related work

Since the 1980s, protocols and standards to validate BP measurement devices have been released. They have provided guidelines for subject requirements, blood pressure distributions, and validation metrics. In terms of subject requirements, a minimum of 85 subjects are needed^24–26^. As to blood pressure distribution, the European society of hypertension has defined accepted ranges of BP in subjects^27^ while other standards bodies^24,25,28^ have defined minimum numbers for samples within different BP ranges. As to validation metrics, most protocols and standards use Mean Error (ME) and Standard Deviation (SD) for evaluation^24,25,27,28^. In 2014, the IEEE established the standard for wearable cuffless BP devices, which first required reporting Mean Absolute Error (MAE) in the validation result^26^. Despite the minor differences between the different standards and protocols, the validation procedure of BP monitoring devices is well-defined. However, none of these standards apply for validation and comparison of the ML based algorithms as they involve learning data. Standards aim to validate existing approaches, whether or not ML based, to ensure the requirements of their claimed intended use. That is, once an ML algorithm is developed and properly evaluated with our proposed benchmark, it must still undergo standard validation afterward to be certified.

Recently, there have been efforts in comparing different ML based BP estimation approaches. Maqsood *et al*.^6^ analyzed handcrafted features from the PPG signal and concluded that time-domain features were more accurate than frequency-domain features. Rather than using the PPG only, in another paper by Maqsood *et al*.^8^, the authors reviewed DL methods that used PPG and Electrocardiogram (ECG) waveforms, which is outside the scope of this paper, as this paper focuses on using PPG signals only. Furthermore, the conclusion that nonlinear models outperformed linear models drawn by Hajj *et al*.^7^ might be limited as it was conducted on specific datasets only. Similarly, papers by Mahmud *et al*.^29^, Athaya *et al*.^22^, and Aguirre *et al*.^12^ showed comparisons between their approaches and others on selected datasets only.

The proposed benchmark in this paper covers a broad variety of datasets with various characteristics so that the conclusions can be more general. The benchmark is also not limited to either handcrafted or automatically extracted features. The validation scheme and evaluation metrics are tailored to correctly compare data-driven ML methods. The datasets and code to prepare the data and validate the results are provided. Open datasets and code enable this benchmark to provide fairly comparable results and allow for reproducibility. Furthermore, it is also extensible by adding new models and new evaluation metrics.

## Results

The proposed standard benchmark is used to compare 11 different state-of-the-art algorithms of three categories, namely, Feat2Lab, Sig2Lab, and Sig2Sig. Results are shown on four publicly available datasets with different characteristics. First, we present the datasets and briefly describe the essential concepts before the following analysis. Then, we analyze the performance of the algorithms within each category and across them. We examine the most relevant features selected by the Feat2Lab algorithms. Finally, we stress the importance of a proper data-splitting strategy with the results of different validation schemes.

### Data characteristics

This study uses four different datasets^14^ briefly summarized in Table 1. The table shows the dataset characteristics, such as data amount before and after preprocessing, demographic information, the continuity property among segments, and the data distribution.

**Table 1:** The table summarizes four datasets used in this study.

Dataset Name	Original Amount	Processed Amount	Demography (%Male & Age)	Sampling Rate (Hz)	Segment Length (s)	Segment Continuity	Validation Strategy	Data Distribution (SBP/DBP)
Sensors^12,30^	Subject: 1196 Record: 5821 Segment: 11642 Duration: ~16 hours	Subject: 1195 Record: 5726 Segment: 11102 Duration: ~15 hours Seg./Sub.: ~9	59.8% 57.1 ± 14.2	125	5	Discrete	5-fold CV	Min.: 81.84/50.07 Max.: 198.66/116.64 Mean: 134.36/65.37 SD: 21.78/10.51
UCI^15,31^	Subject: unknown Record: 11844 Segment: 518036 Duration: ~719 hours	Subject: unknown Record: 10793 Segment: 410596 Duration: ~570 hours Seg./Rec.: ~38	unknown	125	5	Continuous	HOO	Min.: 64.45/50.00 Max.: 199.66/102.18 Mean: 131.57/66.79 SD: 11.16/10.48
BCG^16,32^	Subject: 40 Record: 40 Segment: 3268 Duration: ~5 hours	Subject: 40 Record: 40 Segment: 3063 Duration: ~4 hours Seg./Sub.: ~76	44.5% 34.2 ± 14.5	1000	5	Continuous	5-fold CV	Min.: 71.75/44.47 Max.: 191.07/100.67 Mean: 120.99/67.23 SD: 15.29/9.30
PPGBP^17,33^	Subject: 219 Record: 219 Segment: 657 Duration: < 1 hour	Subject: 218 Record: 218 Segment: 619 Duration: < 1 hour Seg./Sub.: ~3	46.9% 56.9 ± 15.8	1000	2.1	Discrete	5-fold CV	Min.: 80.00/42.00 Max.: 182.00/107.00 Mean: 128.02/71.91 SD: 20.50/11.20

It shows the amount of original (downloaded) data and processed data, demographic information (sex and age), the sampling rate and segment length of each dataset, the continuity property among segments, the applied validation strategy, and the statistics of Systolic Blood Pressure (SBP) and Diastolic Blood Pressure (DBP). Some abbreviations in the table: Subject (Sub.), Segment (Seg.).

**Sensors dataset**^12,30^ is a subset of the MIMIC-III, which includes records of 1195 patients in the intensive care units. PPG and ABP waveforms were collected using Philips CareVue Clinical Information System and iMDsoft MetaVision ICU. As a particularity, the authors kept only two 15 s segments spaced 5 min apart per record. The Sensors dataset has a medium-to-large number of segments and subjects with a high sample variability, a decent ratio of segments per subject, and a discrete data segmentation.

**UCI dataset**, also known as Cuff-Less Blood Pressure Estimation Dataset^15,31^ is a subset of the MIMIC-II Waveform Dataset. MIMIC-II and MIMIC-III come from the same underlying sets of records, sharing conditions, hospitals, and collection devices. However, the Sensors and UCI datasets are different subsets, so they are unlikely to share records. Furthermore, the UCI dataset includes complete records and no limitation of data per record. Originally UCI dataset was released in four different parts without subject information. After preprocessing, it is the biggest dataset with a considerably higher ratio of continuous segments per record.

**BCG dataset** is the bed-based ballistocardiography dataset collected by Carlson *et al*.^16,32^. Signals were recorded from 40 subjects with one record per subject. Four subjects have some previous heart conditions, while the rest were healthy. The data collection was done under Kansas State University IRB protocol #9386, using Finapres Medical Systems Finometer PRO, for the continuous brachial blood pressure, and GE Datex CardioCap 5 for PPG. We resampled the original 1000 Hz signals at 125 Hz and re-scaled the BP signals by a factor of 100 mmHg/volt. BCG dataset is a small to medium-sized set with less data variation given its low number of subjects; its remarkably high ratio of segments per subject; and a narrower BP distribution.

**PPGBP dataset**^17,33^ involves 219 subjects with different cardiovascular diseases, such as hypertension and diabetes. After 10 minutes of rest, one BP reading was recorded per subject with the Omron HEM-7201 device, followed by three 2.1-second PPG segments with the SEP9AF-2 device. Thus, it is the smallest set in the number of segments (613) but with a relatively high number of subjects. The original sampling frequency of 1000 Hz was resampled at 125 Hz.

### A benchmark for machine-learning based non-invasive blood pressure estimation using PPG

Here, we briefly describe the main aspects of our benchmark to understand the following results.

#### Data preprocessing

The four datasets have been preprocessed following the same procedure. First, PPG and ABP signals were aligned based on the maximum cross-correlation and segmented into 5-seconds chunks without overlapping. Then, we remove distorted ABP segments from which it is impossible to identify cardiac cycles or that do not follow reasonable values of amplitudes (30–220 mmHg), pulse pressure (over 10 mmHg), and heart rate at rest (35–140 BPM). From each ABP segment, SBP and DBP labels were extracted by the median of the systolic peaks and the median of the onset and offsets of the cardiac cycles. Finally, the PPG signals have been removed by following the same criteria as in ABP; by eliminating additional distorted signals related to the standard deviation of their peaks and valleys; and by correcting the baseline wander using cubic spline interpolation.

#### ML Algorithms

Our benchmark includes 11 different methods classified into three different categories: Feat2Lab, Sig2Lab, and Sig2Sig. **Feat2Lab** approaches rely on PPG handcrafted features to estimate BP labels. We have considered the most successful PPG features^6,7,19^, comprising time-based, frequency-based, and statistical features. We conducted feature selection based on the mean decrease of the Gini impurity achieved across tree-based ensembles independently trained for SBP and DBP. The features sorted by importance can be selected by a hyperparameter. The Feat2Lab models are classical and popular ML methods, such as Light Gradient Boosting Machine (LightGBM)^34^, Support Vector Regressor (SVR)^35^, Multi-Layer Perceptron (MLP)^36^, Adaptive Boosting (AdaBoost)^37^, and Random Forest (RF)^38^. **Sig2Lab** models directly learn from the PPG signal to output BP labels. Among the Sig2Lab approaches, ResNet^11,39^, SpectroResNet^20^, and MLP-BP^21^ were selected as representative algorithms. The SpectroResNet method consists of a ResNet-GRU architecture for the extraction of temporal and spectro-temporal information. MLP-BP adapted MLP-Mixer neural networks for BP estimation. **Sig2Sig** approaches generate continuous ABP signal from continuous PPG signal. We have considered U-Net^40^, PPG2IABP^12^, and V-Net^23^ in this category. U-Net is the base architecture of several BP estimation approaches^22,41,42^. PPG2IABP^12^ proposed GRU encoder-decoder architecture with an attention mechanism to estimate an ABP’s mean cycle. When implementing previous works, the models that originally used ECG have been adapted to only use PPG. Besides, we are not using any subject calibration or PPG scaling.

#### Validation

We have used 5-fold Cross-Validation (CV) for Sensors, BCG, and PPGBP datasets, while the Hold-One-Set-Out (HOO) strategy was used with UCI datasets. The original UCI dataset was released without subject identification. Due to this and its large number of samples, we decided to follow the HOO strategy. In our validation strategies, the data is not split into folds, as usual, with a uniform probability distribution, because it would lead to different examples of the same subject in different folds, i.e. information leakage, and there is a risk that one or more folds have few or no examples of underrepresented BP labels (very high or low BP values). To mitigate these potential issues, the data are split considering the subjects and following a stratified partitioning procedure for multi-label data^43,44^.

#### Evaluation metrics

The performance of the different algorithms is assessed on the estimation of both SBP and DBP. Feat2Lab and Sig2Lab output BP labels directly, while for Sig2Sig, the estimated labels are extracted from the predicted ABP by identifying the systolic peaks, onset, and offset of each cardiac cycle. Thus, we consider the commonly used metrics of MAE, ME, and SD. Besides, we propose MASE as the main evaluation metric. MASE is computed as the ratio of the model’s MAE and the naïve MAE result. The naïve predictions are the mean of the SBP and DBP labels of the training dataset.

### Comparison of machine learning based blood pressure estimation approaches

**Fig. 2:**
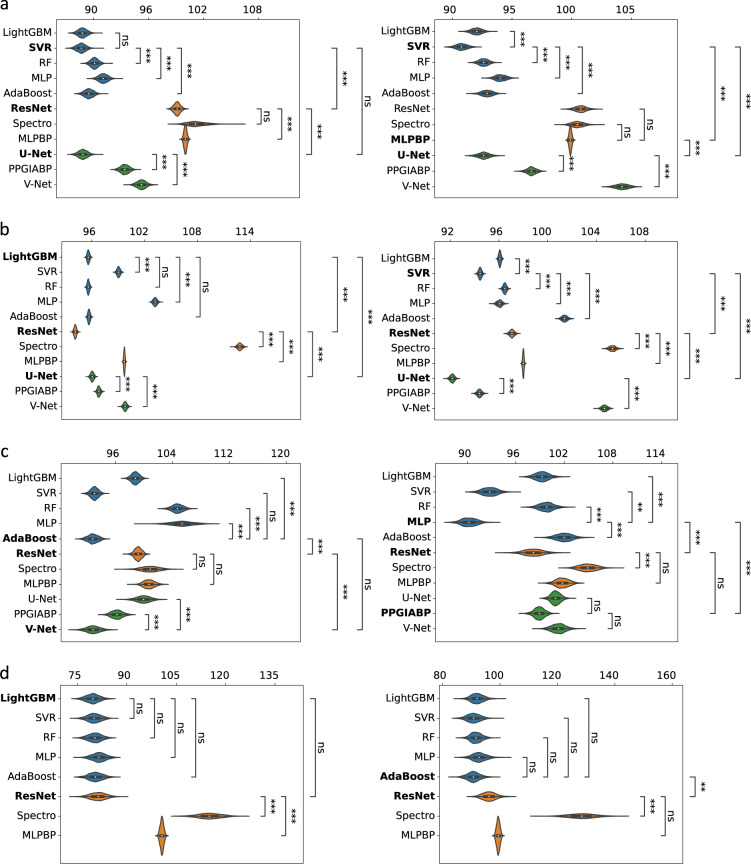
MASE results for SBP (left) and DBP (right) with confidence intervals extracted with bootstrapping of (**a**) Sensors, (**b**) UCI, (**c**) BCG, and (**d**) PPGBP. The significance lines show the pairwise comparison of the best model against the same category models (inside) and across categories (outside). The significance (*a* = 0.001(***), 0.01(**), 0.05(*)) is measured by assessing if the 1-*a* confidence intervals of the models’ difference contain 0. ‘ns’ stands for not significant. Bonferroni’s correction is used for these multiple comparisons. We highlight with bold type the best result within each category.

Now, we compare the different representative ML models from the three categories. Table 2 shows the performance results of the 11 algorithms grouped into three categories. The result of the best-performing algorithm for each metric and each dataset appears in bold. The results of U-Net are omitted in the PPGBP dataset due to the lack of ABP for training this model. As a scale invariance metric, MASE brings both SBP and DBP errors on a comparable scale and helps the comparison across different datasets. Besides, MASE does not require extra information from the data distribution to be interpretable unlike absolute metrics, such as the MAE and ME ± SD. Therefore, we best summarize the model performance using MASE in Fig. 2. Figure 2 demonstrates the model comparison by showing the confidence intervals extracted with bootstrapping^45^, while the significance lines are computed by pair-wise algorithm comparisons. The analysis of the results is conducted within each category and across them, as follows:

**Table 2:** Performance of the ML algorithms grouped in three categories on four datasets.

Sensors dataset						
	SBP			DBP		
	MAE	ME ± SD	MASE (%)	MAE	ME ± SD	MASE (%)
Naïve	17.61	−0.01 ± 21.82	100.00	8.27	0.00 ± 10.53	100.00
LightGBM	15.63	**−0.05** ± **19.64**	88.76	7.61	**−0.02** ± **9.82**	92.04
SVR	**15.60**	−0.00 ± 19.68	**88.62**	**7.50**	−1.45 ± 9.81	**90.76**
RF	15.86	−0.12 ± 19.85	90.08	7.66	−0.03 ± 9.86	92.63
MLP	16.03	−0.50 ± 20.10	91.03	7.77	−0.19 ± 10.04	94.05
AdaBoost	15.75	−0.06 ± 19.77	89.45	7.68	−0.27 ± 9.96	92.91
ResNet	17.46	−0.12 ± 21.70	99.15	8.33	−2.51 ± 10.78	100.76
SpectroResNet	17.83	0.90 ± 28.05	101.28	8.31	0.13 ± 11.08	100.52
MLPBP	17.61	0.01 ± 21.86	100.03	8.26	−0.02 ± 10.51	99.90
U-Net	15.64	−1.16 ± 19.64	88.82	7.66	−0.45 ± 9.93	92.62
PPGIABP	16.45	−3.23 ± 20.41	93.40	7.99	−0.31 ± 10.28	96.64
V-Net	16.77	−7.06 ± 19.95	95.21	8.62	3.52 ± 9.82	104.26
**UCI dataset**						
Naïve	17.62	0.57 ± 21.86	100.00	8.55	−0.65 ± 11.40	100.00
LightGBM	16.85	1.53 ± 20.62	95.60	8.21	−0.22 ± 11.00	96.07
SVR	17.45	2.10 ± 21.25	99.02	8.07	−1.02 ± 11.06	94.46
RF	16.85	1.26 ± 20.67	95.60	8.25	**0.03** ± **11.08**	96.48
MLP	18.18	3.67 ± 21.92	103.18	8.21	0.90 ± 11.02	96.05
AdaBoost	16.86	1.19 ± 20.86	95.68	8.67	0.23 ± 11.72	101.39
ResNet	**16.59**	−3.90 ± 20.65	**94.12**	8.30	−4.80 ± 10.84	97.06
SpectroResNet	19.88	3.99 ± 24.20	112.78	9.00	0.85 ± 12.16	105.31
MLPBP	17.57	−3.56 ± 21.84	99.69	8.38	−1.68 ± 11.30	98.00
U-Net	16.93	**0.06** ± **20.92**	96.04	**7.88**	−2.46 ± 10.80	**92.17**
PPGIABP	17.06	0.20 ± 20.99	96.79	8.07	0.25 ± 10.99	94.41
V-Net	17.58	−9.28 ± 20.53	99.78	8.95	3.90 ± 10.66	104.67
**BCG dataset**						
Naïve	12.30	−0.19 ± 16.67	100.00	7.91	−0.11 ± 9.96	100.00
LightGBM	12.15	−1.12 ± 16.78	98.80	7.84	−0.04 ± 10.29	99.19
SVR	11.45	**−0.79** ± **15.56**	93.07	7.34	0.01 ± 9.88	92.75
RF	12.88	−1.46 ± 17.75	104.72	7.89	−0.01 ± 10.44	99.77
MLP	12.98	−0.27 ± 16.35	105.50	**7.14**	**0.03** ± **9.28**	**90.24**
AdaBoost	**11.42**	−2.50 ± 16.44	**92.84**	8.06	−0.33 ± 10.73	101.91
ResNet	12.20	−0.67 ± 16.69	99.20	7.76	−4.75 ± 8.98	98.13
SpectroResNet	12.41	1.34 ± 16.49	100.93	8.30	1.22 ± 10.41	104.91
MLPBP	12.39	−1.02 ± 16.77	100.75	8.05	−0.32 ± 10.31	101.81
U-Net	12.30	1.32 ± 16.42	99.98	7.98	−0.09 ± 10.45	100.94
PPGIABP	11.66	−2.52 ± 15.95	94.76	7.78	−1.67 ± 9.88	98.37
V-Net	11.42	−3.89 ± 14.84	92.89	8.01	−1.27 ± 10.10	101.25
**PPGBP dataset**						
Naïve	16.38	−0.02 ± 20.52	100.00	8.85	0.00 ± 11.20	100.00
LightGBM	**13.06**	**0.00** ± **16.65**	**79.76**	8.16	**−0.04** ± **10.30**	92.18
SVR	13.15	−0.64 ± 17.05	80.29	8.04	−0.22 ± 10.14	90.90
RF	13.17	0.02 ± 16.81	80.42	8.12	0.19 ± 10.17	91.76
MLP	13.38	−0.13 ± 17.09	81.69	8.21	−0.16 ± 10.40	92.77
AdaBoost	13.22	−0.56 ± 16.95	80.72	**8.04**	−0.16 ± 10.25	**90.84**
ResNet	13.62	−1.85 ± 17.45	83.18	8.61	−2.17 ± 10.81	97.33
SpectroResNet	18.87	−6.26 ± 23.76	115.18	11.38	−5.22 ± 14.59	128.60
MLPBP	16.49	−0.81 ± 20.66	100.68	8.80	−0.52 ± 11.22	99.41

#### Feat2Lab category

Overall, SVR and LightGBM are the best models among the Feat2Lab, since they frequently achieve the first or second-best results in terms of MASE. In particular, SVR, closely followed by LightGBM, significantly outperforms the rest in the Sensors dataset (Fig. 2a). In Fig. 2b related to UCI, LightGBM, and SVR are again the best in SBP and DBP, respectively. In contrast, Adaboost and MLP result better in SBP and DBP of the BCG dataset, while SVR is the second best as shown in Fig. 2c. Although Fig. 2d shows no significant difference in PPGBP’s results, LightGBM and Adaboost stand out in SBP and DBP, respectively. Despite some absence of significant differences, the LightGBM is considered more efficient in its training and inference, especially with large datasets^34,46^.

#### Sig2Lab category

ResNet is the best model among the Sig2Lab, achieving the lowest MASE in all datasets, excluding DBP of Sensors. In Fig. 2a (Sensors) and 2c (BCG), the results of the different Sig2Lab models are very similar, especially for ResNet and MLPBP. While for UCI and PPGBP datasets, ResNet significantly outperforms the rest of the models, and SpectroResNet performs worse than usual as shown in Fig. 2b,d.

#### Sig2Sig category

In Sensors and UCI datasets, U-Net is significantly the best model, followed by PPGIABP, and lastly, V-Net. In contrast, V-Net significantly outperforms the rest for SBP estimation of the BCG dataset, and PPGIABP is slightly better for DBP as shown in Fig. 2c. Therefore, we consider U-Net as the best model among Sig2Sig algorithms.

#### Across categories

Feat2Lab approaches achieve better results for the smaller datasets, BCG and PPGBP. However, there are some cases where the best results of Feat2Lab, Sig2Lab, and Sig2Sig are comparable. For the BCG dataset, the first models of Feat2Lab and Sig2Sig (Adaboost and V-Net) show comparable performance for SBP estimation in Fig. 2c. In the Sensors dataset, SVR and U-Net show similar SBP results in Fig. 2a. For the largest dataset (UCI), ResNet and U-Net are the best models for SBP and DBP, respectively. Thus, Sig2Lab and Sig2Sig approaches can outperform Feat2Lab models, but they require considerably large datasets.

### Feature importance in *Feat2Lab* models

As previously mentioned, we have considered the most popular features for Feat2Lab models: point/time-based, which comprise elapsed times, amplitudes, areas, and width between points of interest of the cardiac cycle; frequency-based features; and statistical features, which include histograms, Slope Deviation Curve (SDC), Signal Quality Index (SQI), and indices features. Given a large number of features, we conducted feature selection based on the Gini impurity independently for SBP and DBP of each dataset. Thus, we assess the most relevant individual features and subgroups.

**Fig. 3:**
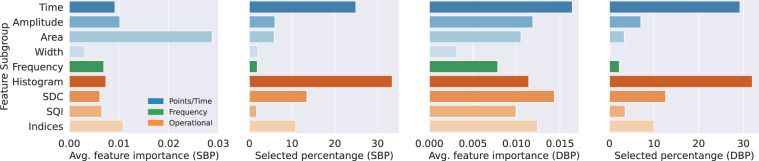
Relevance of the selected features divided into subgroups of the main three families: Point/time-based, Frequency-based, and Operational/Statistical features. Feature relevance is computed in two ways: the average feature importance among the features selected by the models, and the percentage of each subgroup among the selected features.

Overall, the three highest-ranked features are *T*_*s_e*_, *T*_*s_z*_, and *vpg*_*z*_, which are the elapsed time from the systolic peak to the dicrotic notch (*e*) and the diastolic rise (*z*), and the amplitude of the first derivative (*vpg*) at point *z*. Besides, we show the relevance of each feature subgroup in Fig. 3. Among the features selected by the algorithms, the feature groups with the largest percentage are histogram-based and time-based features for both SBP and DBP. However, these can be biased by the larger number of these features. When taking the average feature importance into account, the time-based features remind as one of the most important, while the importance of the histograms is reduced. Therefore, we can ensure that the time-based features are highly relevant to the models. Besides, some of the area-based and SDC features are also relevant for SBP and DBP, as indicated by their average feature importance. Frequency-based and width-based features are the least relevant features with the smallest percentage and average importance. Moreover, width-based features share similar information to elapsed time features, which justifies their low relevance to the models.

### Data splitting: subject information and skewed blood pressure distributions

General ML model development includes splitting a dataset into training, validation, and test sets. BP datasets usually have multiple records corresponding to the same human subject. Besides, the SBP and DBP frequently exhibit skewed distributions. When splitting the data into folds with a uniform probability distribution, a common practice in ML, it would lead to examples of the same subject in different folds, i.e. information leakage, and to the risk of having folds with few or no samples of underrepresented BP labels. Here, we analyze the impact of overlooking these issues.

**Fig. 4:**
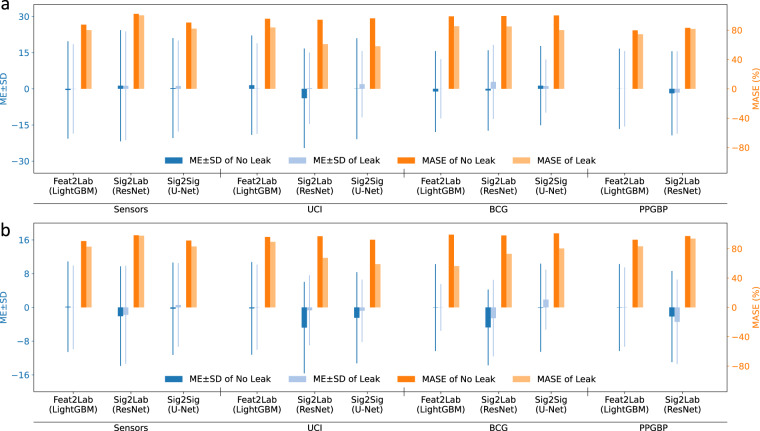
The experiment compares with and without leaking subject information in different sets for **(a)** SBP and **(b)** DBP. In the leaked datasets, the percentage of samples of the test set that share subject with the training set are 100% in Sensors and BCG, 99.5% in UCI, and 94.6% in PPGBP.

When different segments of the same subjects are simultaneously in different partitions, the results can be misleading and over-optimistic. Models may rely mainly on subject-specific characteristics to estimate their BP. This is more pronounced with consecutive segments of the same subject since their BP values do not change much. Figure 4 exemplifies this by comparing the performance of a model of each category on the different datasets, with or without this issue (Leak or No Leak, respectively). The leaked datasets were split into different folds with a uniform distribution. As shown, all three models have better results in the leaked scenario than the no-leaked scenario regardless of the evaluation metric used. The difference in performance is more significant for UCI and BCG, which have multiple consecutive segments, 32 and 64 segments on average, respectively. In UCI and BCG datasets, the MASE of SBP and DBP decrease from values around 98–92% to below 60% in some cases. Besides, the significant drop is also shown in the SD metric. This is a common mistake in BP estimation research, where some practitioners considered it as a way of personalization or calibration^12,20^, and sometimes, it is not clear whether some proposals fall into this error^20^.

**Fig. 5:**
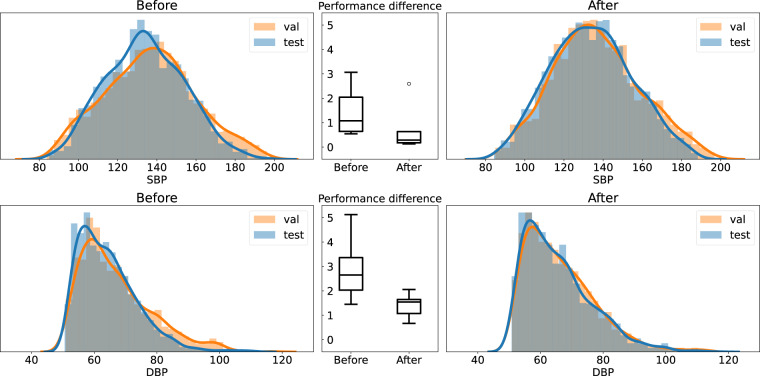
Examples of before and after maintaining the training/validation/test sets distribution are shown in the histogram. The differences between the MASE performance of the validation and the test sets are shown in box plots.

We also emphasize the importance of accounting for the skewed distributions of SBP and DBP. Figure 5 shows the difference in the MASE between validation and testing **before** and **after** maintaining the SBP and DBP distribution. We picked the Sensors dataset to demonstrate this because the other datasets lack subject information or are too small. In Fig. 5, we can see a larger mean and standard deviation in the MASE difference of validation and test sets before maintaining the distribution. This implies that the results in validation will not transfer correctly to test, because some folds will have fewer examples of extreme BP labels as shown in Fig. 5.

## Discussion

In this paper, we have presented a standardized benchmark for ML and DL based non-invasive BP estimation approaches using the PPG waveform. Our benchmark includes four different datasets with a wide variety in the number of subjects and segment continuities. We have extensively described three learning paradigms for BP estimation (Feat2Lab, Sig2Lab, and Sig2Sig). Furthermore, we have defined the standard evaluation metrics and proposed using MASE to compare performance across datasets. Cross-validation strategies have been adapted to the problem singularities, ensuring the correct training and tuning of the ML models. We have empirically compared 11 different approaches of the three paradigms, setting the baselines for future model development and comparisons. We have analyzed the importance of the feature groups used in Feat2Lab approaches. Besides, we have shown the impact of overlooking important factors when preparing validation folds, such as BP skewed distribution and multiple samples per subject. This study enables reproducibility and fair comparison among different BP estimation proposals with shared datasets and code.

Next, we discuss the main conclusions of our experiments giving some interpretations and suggestions when addressing a BP estimation problem with PPG using ML and DL approaches:

- When splitting the data into validation folds, the skewed BP distribution and subject information leakage are commonly overlooked in BP research^11,12,20^, even discussing the latter as a calibration strategy^20^. We have empirically shown how overlooking them leads to misleading and over-optimistic results. Therefore, we have shared a data splitting that considered these particularities.
- Comparing the different approaches within each category, SVR and LightGBM have the best performance among the Feat2Lab models, while the latter, as a versatile ML model, enables great fitting and efficiency in different scenarios. ResNet significantly outperforms the rest of the automatic feature extractors. Besides, U-Net is the best model among the Sig2Sig approaches.
- Across different families of approaches, Feat2Lab models still are very competitive, especially for medium and smaller datasets. ResNet and U-Net outperform the rest of the algorithms in the largest dataset (UCI). Sig2Lab approaches have the potential to outmatch Feat2Lab proposals and eliminate the arduous and error-prone task of hand-crafted feature extraction. Sig2Sig methods, leading with the U-Net architecture, are always preferred since they are available to estimate ABP. However, the need for massive amounts of invasive ABP waveforms makes its training and implementation challenging.
- Analyzing the importance of the features selected by Feat2Lab approaches, we have concluded that the elapsed times between PPG’s points of interest are the most relevant features for both SBP and DBP. In particular, the times related to the dicrotic notch and the diastolic rise of the PPG cardiac cycle have ranked very high. Besides, area and SDC features are valuable to estimate SBP and DBP, respectively. On the other hand, width-based and frequency-based features are the least relevant.
- MASE has proven useful for the comparison and interpretation of model performance across different BP datasets. For instance, given the SVR’s MAE of 15.60 in the Sensors dataset and 11.42 in the BCG dataset, one may conclude that the model trained with BCG performs significantly better. This is not necessarily true, as it is natural to obtain smaller errors in the BCG dataset due to its narrower BP range. Looking at the MASE of 88.62% in Sensors and 92.84% in BCG, we can realize that the performance of the model trained with Sensors data is slightly better.
- As shown by our results, PPG-based proposals for non-invasive BP estimation still require substantial research to meet the requirements of medical validation standards. Other physiological signals, such as ECG, or individual calibration might help to reach those accuracy standards, but it reduces applicability, usability, and portability. We hope our benchmark serves as a baseline and eases the model comparison for future research and proposals.

## Methods

This section provides the design of our benchmark for ML and DL based BP estimation approaches. First, we describe the data preprocessing and preparation steps. Second, we detail the ML and DL based BP estimation approaches and categorize them into three categories: Feat2Lab, Sig2Lab, and Sig2Sig. Then, we explain the adapted validation strategies to train and tune the ML/DL models. We define the evaluation metrics used in our benchmark. Lastly, we describe the procedure followed to tune the hyperparameters of ML and DL models.

### Data preparation and preprocessing

**Fig. 6:**
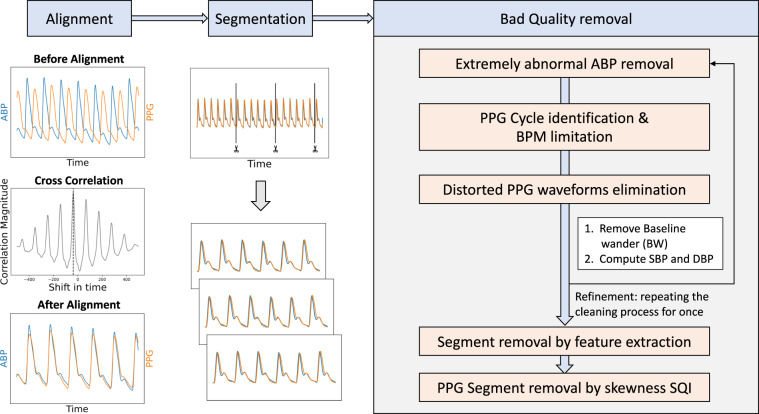
Data preprocessing pipeline.

**Fig. 7:**
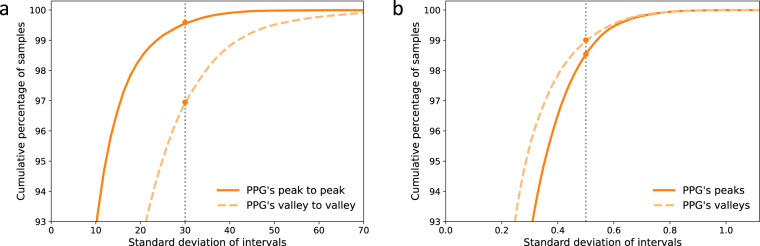
Cumulative percentage plots of the standard deviations of PPG’s **(a)** intervals and **(b)** amplitudes for the UCI dataset.

**Fig. 8:**
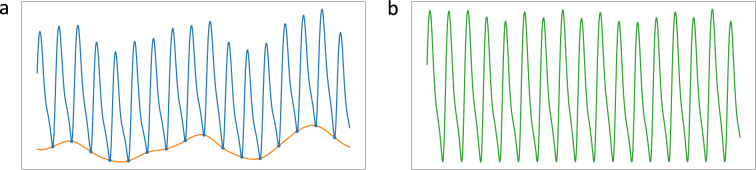
Example of Baseline Wander correction using CSI. **(a)** Original waveform in blue and its baseline in orange. **(b)** Corrected waveform.

Data preparation and preprocessing are crucial to ML and DL model training as they clean noise and signal artifacts to avoid perturbed modeling. This process has been designed as a standard and common procedure for all the presented datasets. Figure 6 shows an overview of the whole data preparation and preprocessing process, while Table 3 lists the data amount in every cleaning step. First, the procedure aligns the signals (PPG & ABP) of each record available using cross-correlation. The PPG signal usually has a certain delay to the ABP signal due to the difference in the extraction points^23^. This delay might affect the learning and estimation of ML and DL approaches, particularly those estimating the ABP waveform^22,23^. The alignment shift is set as the maximum cross-correlation magnitude, limited to a maximum of 1 second to avoid an excessively unrealistic shift. Once aligned, each record is segmented into 5-seconds chunks without overlapping. The following preprocessing steps aim at removing poor quality signals caused by numerous factors, such as noise, movement and respiration artifacts, outliers, and extreme cases:

- **Extremely abnormal ABP removal:** Although ABP is the gold standard to measure blood pressure, it is not spared from errors and disturbances. To avoid training and testing on erroneous signals, we remove extremely distorted ABP segments from which it is impossible to identify cardiac cycles or that do not follow reasonable values of amplitudes (30–220 mmHg), pulse pressure (over 10 mmHg), and heart rate at rest (35–140 Beats Per Minute (BPM)).
- **PPG cycle identification and BPM limitation:** The cardiac cycles are delimited by an initial valley, the systolic peak, and a second valley. Segments with missing or excessive valleys or peaks are excluded. Additional segments are removed if their heart rates are abnormal for adults at rest (35–140 BPM).
- **Distorted PPG waveforms elimination:** Additional distorted PPG waveforms are identified by high standard deviations of the peak-to-peak and valley-to-valley intervals as well as their amplitudes^29^. We eliminate any segments whose standard deviations exceed certain thresholds. These were set by examining the waveforms and cumulative percentage plots of these statistics. Figure 7 shows the cumulative percentage plots of the mentioned statistics and vertical lines of the chosen thresholds for the UCI dataset.
- **Baseline Wander (BW) removal:** BW is a low-frequency artifact commonly caused by respiration and movement. To correct it, we estimate the baseline using Cubic Spline Interpolation (CSI) on the valleys of the segments^47^. Then, the estimated baseline is subtracted from the original segment as shown in Fig. 8.
- **Refining and SQI:** Finally, we reiterate the aforementioned cleaning process to ensure high signal quality standards. We perform the feature extraction explained in the following section, eliminating the segments with which the extraction process fails. Lastly, we also exclude those signals with SQI skewness below 0^48^.

**Table 3:** The table details the amount of data in preprocessing steps.

Step	Criterion	Sensors dataset	UCI dataset	BCG dataset	PPGBP dataset
0	Ori: # subjects/records/segments	1196/5821/11642	-/11844/518036	40/40/3268	219/219/657
1	Del: # segs. with ABP > 220 or < 30 mmHg	4	16	0	—
	Del: # segs. with ABP’s BPM > 140 or < 35	1	2205	0	—
	Del: # segs. with pulse pressure < 10 mmHg	0	456	0	—
	Kept: # subjects/records/segments	1192/5821/11637	-/11788/515359	40/40/3268	219/219/657
2	Del: # segs. with no peaks or valleys found	1	35	0	11
	Del: # segs. removed by p2p distance (BPM)	34	4019	34	0
	Del: # segs. removed by v2v distance (BPM)	43	4458	36	0
	Kept: # subjects/records/segments	1196/5808/11586	-/11710/509453	40/40/3213	217/217/646
3	Del: # segs. with PPG distortion	373	29203	74	0
	Kept: # subjects/records/segments	1195/5751/11213	-/11581/480250	40/40/3139	217/217/646
4	Del: # segs. show bad quality after BW removal	10	13492	58	4
	Kept: # subjects/records/segments	1195/5741/11139	-/11499/466758	40/40/3081	219/219/642
5	Del: # segs. failed in feature generation	33	48422	10	2
	Kept: # subjects/records/segments	1195/5726/11106	-/11057/418336	40/40/3071	219/219/640
6	Del: # segs. With skewness SQI < 0	4	7740	8	21
	Kept: # subjects/records/segments	1195/5726/11102	-/10793/410596	40/40/3063	218/218/619

The definition of each step is as follows: Step 0 - alignment and segmentation; Step 1 - Extremely abnormal ABP removal; Step 2 - Cycle identification and BPM limitation; Step 3 - Distorted waveforms elimination; Step 4 - Refinement after baseline wandering; Step 5 - Segment removal by feature extraction; Step 6 - Segment removal by skewness SQI. The terms “Ori”, “Del” and “Kept” refer to the original amount, deleted amount, and kept amount, respectively. One should notice that every segment could meet several removal criteria simultaneously in every step.

### Feat2Lab: From PPG waveform features to BP labels

Feat2Lab approaches engineer meaningful representations of PPG waveforms to help ML regression models learn the relation between PPG and BP . This paper has considered the most successful and popular features of PPG and its derivatives^6,7,49^. We have categorized them into three groups: points-of-interest and time-based features, frequency-based features, and finally, operational and statistical features .

**Fig. 9:**
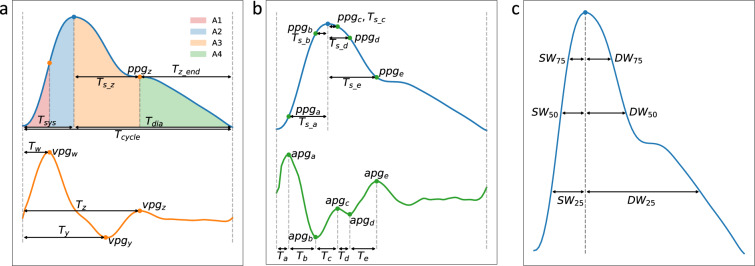
Extracted features related to **(a)** VPG’s points, **(b)** APG’s points, and **(c)** the width of the systolic and diastolic phases (SW & DW) at a given elevation of the systolic peak. Amplitude features of PPG and APG are represented, for point *e* as an example, with *ppg*_*e*_ and *apg*_*e*_ respectively. Time-based features measure the time passed between two points, for instance, *T*_*s_z*_ is the time between *s* and *z*. Areas under the curve (a) are computed in different cycle phases including the areas of systole *A*_*sys*_ = *A*1 + *A*2, and diastole *A*_*dia*_ = *A*3 + *A*4.

**Points-of-interest and time-based features** characterize the signal morphology by extracting particular points from the PPG cardiac cycle and its derivatives^49^: the systolic peak from PPG; *w*, *y*, *z* from the first derivative (VPG); and *a*, *b*, *c*, *d*, *e* from second derivative (APG). Then, different features are computed as shown in Fig. 9: (1) **Amplitudes** of PPG, VPG, and APG for each point, (2) **Elapsed Times**, and (3) **Areas** under the PPG curve. In addition, we have considered the (4) **Widths** of the systolic and diastolic phases (SW & DW) at 25%, 50%, and 75% of the systolic peak amplitude^7^, as shown in Fig. 9c. The sum and ratio of DW and SW at the same percentage are considered additional features.

**Frequency-based features** are extracted from the information of the Fast Fourier transform (FFT) of the PPG waveform. We have included the most dominant frequency, its magnitude, and the average magnitude nearby it^20,50^.

**Operational and statistical features** characterize the PPG cardiac cycle with distribution information, indices, and features combinations: (1) **Histogram features** are the density values of a 5-bin histogram for the systolic phase, and a 10-bin histogram for the diastolic phase in PPG, VPG, and APG. (2) **Slope Deviation Curve (SDC) features** are the deviation of the systolic upstroke waveform and the diastolic falling waveform from their corresponding mean slope curves^50^. (3) **SQI features** are Skewness and Kurtosis of the PPG cardiac cycle. (4) **Indices features** are the Aging Index (AI) and three other indices (*I*_*bd*_, *I*_*bcda*_, and *I*_*sdoo*_)^51^.

Given a large number of features, we conduct feature selection based on tree-based ensembles. We train fully-grown RF^38^ and Extra-Trees^52^ with 500 trees independently for SBP and DBP. The feature importance is the normalized mean decrease of the Gini impurity achieved across the ensembles. Thus, the features sorted by their importance can be selected by a hyperparameter of the percentage of desired features. With these features and the selection procedure, we can use any ML regressor algorithm to estimate blood pressure. We have considered the most popular models^6,7,53^ such as LightGBM^34^, SVR^35^, MLP^36^, AdaBoost^37^, and RF^38^.

### Sig2Lab: From PPG signal to BP labels

Aside from extracting features via handcrafted methods, Convolutional Neural Network (CNN) serves as an automatic feature extractor that could capture signal morphological information. Considering that expert-knowledge-based feature extraction techniques are time-consuming and susceptible to noisy signals, CNN-based models have gained significant interest in PPG signal processing^9,11^. Among the CNN-based models, ResNet^39^ has shown its ability across multi-dimensional signals and is commonly used in PPG feature extraction. For example, Schrumpf *et al*.^11^ compared the BP estimation performance of different neural network architectures, including AlexNet^54^, ResNet, and their proposed CNN-LSTM architecture. They found that ResNet achieved the lowest MAE in both SBP and DBP. Slapničar *et al*.^20^ proposed a ResNet-GRU architecture, called SpectroResNet, to extract the temporal information with residual blocks and spectro-temporal information from PPG’s spectrogram with Gated Recurrent Units (GRU). In another deep learning architecture comparison work^9^, Paviglianiti *et al*. found that ResNet followed by three Long Short Term Memory (LSTM) layers could achieve the best performance. Other deep learning architectures have been applied to estimate BPs, such as MLP-BP^21^–a model that adapts MLP-Mixer neural networks. In this benchmark, we have used ResNet, SpectroResNet, and MLP-BP as the representative algorithms of the Sig2Lab category.

### Sig2Sig: From PPG signal to ABP signal

In addition to intermittent BP measurements, continuous BP monitoring indicates the reactions of the cardiovascular system, which allows physicians to tailor treatment or predict heart failure^55^. In recent years, several engineering works aimed to estimate ABP signals from PPG signals with Recurrent Neural Network (RNN) based models, such as LSTM and GRU, and CNN-based models.

For example, Harfiya *et al*. created an LSTM-based autoencoder for sequence-to-sequence learning. They first trained an autoencoder to reconstruct the PPG waveform input and then further trained the decoder for constructing the ABP waveform^56^. Aguirre *et al*. proposed PPG2IABP, a GRU encoder and decoder network followed by MLP to predict the next value of a target sequence (in this case, ABP signal) given a source sequence (in this case, PPG signal)^12^.

As for the CNN-based models, U-Net consists of a contracting path and an expansive path with bypass connections in between to prevent the loss of border pixels in every convolution^40^. Several works^22,41,42,57^ propose to use U-Net architecture to estimate ABP from PPG due to its capability of signal-to-signal translation. Similarly, Hill *et al*.^23^ proposed a V-Net architecture for the estimation of ABP. Due to their promising and common use, we have considered U-Net^40^, PPG2IABP^12^, and V-Net^23^ as the representative algorithms of the Sig2Sig category in this benchmark paper. Since SBP refers to the maximum pressure while DBP is the minimum pressure within one complete cardiac cycle^58^, they can be extracted from the estimated ABP with peak and valley detection methods afterward.

### Validation strategies

BP estimation is not a standard regression problem. For instance, the data points of the BP datasets are not completely independent of each other, since many segments come from the same subject with very similar information. In addition, there are two targets, SBP and DBP, which are more akin to a multi-task or multi-output regression problem^59^. Finally, the distributions of SBP and DBP are often skewed, as extreme BP is much rarer, which makes it an imbalance regression problem^60^.

These differences must be considered to correctly partition the data during the cross-validation. For example, cross-validation strategies often shuffle the data before partitioning, which may lead to segments of the same subject simultaneously occurring in the training, validation, and test sets. This would result in the breakdown of independence between sets, and potentially lead to unrealistically good results. Moreover, due to the imbalanced distribution, random data partitioning could lead to rare cases missing in the test set.

To avoid these problems, we propose a new procedure for splitting BP data that keeps all samples from the same subject in the same set and the original distribution of SBP and DBP. Maintaining the distributions is not trivial with two different targets and the subject constraint. First, we encode the SBP and DBP values into four classes. The classes of SBP are (1) below 100 mmHg, (2) between 100 mmHg and 140 mmHg, (3) between 140 mmHg and 160 mmHg, and (4) over 160 mmHg. The classes of DBP are (1) below 60 mmHg, (2) between 60 mmHg and 80 mmHg, (3) between 80 mmHg and 100 mmHg, and (4) over 100 mmHg. Then, we count the frequencies of the class combinations (16 classes) for each subject. Thus, we consider the BP label distributions of each subject separately. Finally, we split the subjects with their label distributions into K folds by iterative stratification for multi-label data^43,44^. This partitioning strategy is applicable for K-fold CV and HOO.

### Evaluation metrics

Following the BP standards^25,26,61^, we strongly suggest that researchers report these three metrics simultaneously: the MAE, the ME, and its SD. For the ML pipeline, researchers should gather all the predictions from every fold first and then compute the metrics. The definition of ME is the mean value of the differences as shown in Eq. 1:

1 $$ME=\frac{1}{n}\times \mathop{\sum }\limits_{i=1}^{n}Dif{f}_{i}$$

where *n* is the number of Determinations or Predictions (PREDs) in engineering terms, i is the index of PREDs, while $Dif{f}_{i}=\left({P}_{PRE{D}_{i}}-{P}_{RE{F}_{i}}\right)$ denotes the difference between the *i*^*th*^ pair of blood pressure values (predicted blood pressure - reference blood pressure). SD is the standard deviation of differences as shown in Eq. 2. MAE, on the other hand, is defined as the mean of absolute differences as illustrated in Eq. 3.

2 $$SD=\sqrt{\frac{1}{n-1}\times \mathop{\sum }\limits_{i=1}^{n}{\left(Dif{f}_{i}-ME\right)}^{2}}$$

3 $$MAE=\frac{1}{n}\times \mathop{\sum }\limits_{i=1}^{n}\left|Dif{f}_{i}\right|$$

Besides, comparing the performance of different algorithms is more difficult without fixed BP datasets. The MASE metric^18^ was proposed in time series forecasting to mitigate this issue by scaling the MAE of model predictions with the MAE of the Naïve estimations as shown in Eq. 4. We propose using MASE as the standard BP evaluation metric. We define the Naïve predictions as the mean SBP or DBP of the training set. Along with the Naïve result, the MASE metric is scale-independent and easy to interpret, allowing the comparison of various algorithms across different datasets.

4 $$MASE=\frac{MAE}{MA{E}_{Naive}}$$

### Hyperparameter tuning

Training and hyperparameter tuning were done using nested 5-fold CV, stratified by subject SBP and DBP, except for the UCI dataset with HOO. We tuned ML models by grid searching the parameter-search-space shown in Table 4 and monitoring the MAE performance of validation sets. For the DL models, we used the Mean Squared Error (MSE) as the loss function, the Adam optimizer, and early stopping with the patience of 15 epochs in the validation loss. Their hyperparameters were greedily searched using the Optuna Toolkit^62^ to monitor the MAE performance. Table 4 lists the tuned hyperparameters.

**Table 4:** Parameter-search-space for ML and DL parameters tuning.

Category	Algorithm	Parameter
Feat2Lab	Feature selection	Rate: [0.05, 0.1, 0.15, 0.2, 0.25, 0.3, 0.4, 0.5, 0.7, 0.9, 1.0]
	SVR	Kernel: rbf C: [1.0, 5.4, 10, 100, 170, 1001] Gamma: [0.001, 0.008, 0.1, 0.7, 1] Epsilon: [0.0003, 0.007, 0.01, 0.05, 0.1, 0.15, 0.2]
	MLP	Layer: [[32], [64], [256], [256, 64], [512, 64]]
	AdaBoost	Trees: [5, 10, 50, 100, 150, 200] Maximum depth: [1, 3, 5, 8, None] Minimum samples per leaf: [5, 25, 50]
	RF	Trees: [10, 50, 100, 150, 200, 300, 400] Maximum depth: [1, 3, 5, 8, None] Minimum samples per leaf: [5, 25, 50] Sampling rate: [0.5, 0.7, 0.9] Column sampling per split: [0.3, 0.7, 1.]
	LightGBM	Trees: [10, 50, 100, 150, 200, 300, 400] Learning rate: [0.01, 0.05, 0.1] Maximum depth: [1, 3, 5, 8, None] Minimum samples per leaf: [5, 25, 50] Sampling rate: [0.5, 0.7, 1.]
Sig2Lab	ResNet	Channel: [32, 64, 128, 256] Kernel size of the first conv. layer: [5, 9, 11, 15] Kernel size of residual blocks: [3, 5] Amount of residual blocks: [4, 8, 10]
	SpectroResNet	N. dft, N. hop: [16, 64] Kernel sizes: [[8, 5, 3], [8, 5, 5, 3]] Amount of residual blocks: [4, 8, 10]
	MLPBP	Depth: [4, 6, 8] Dropout: [0.1, 0.2] Token & channel dimension: [256, 512]
Sig2Sig	U-Net	Channel: [8, 16, 32, 64, 128] Layer: [[2, 2], [2, 3, 2], [2, 2, 2], [2, 2, 2, 2]]
	PPGIABP	Hidden size of GRU layers: [4, 8, 10]
	V-Net	Layer: [[2, 2], [2, 2, 2], [1, 2, 3], [1,2,3,3]]

The Layer parameter indicates the number of layers stacked in each depth block. For example, [2, 3, 2] defines the U-Net architecture with 3 depth blocks with 2, 3 and 2 CNN layers, respectively.

## Data Availability

The four datasets used in this paper are available via Figshare^14^. We provide the split datasets where the sensors, BCG, and PPGBP datasets are split into 5 folds, and the UCI is in 3 folds. The purpose of this is to enable researchers to compare their methods under the same split datasets.

## Code availability

The data preprocessing scripts and machine learning algorithm are publicly available via GitHub at https://github.com/inventec-ai-center/bp-benchmark. The custom code used for data visualization is available from the corresponding authors upon request.
